# Archaeobotanical investigations at high-elevation sites of the Pamir Mountains and fergana foothills

**DOI:** 10.1016/j.isci.2025.114349

**Published:** 2025-12-05

**Authors:** Kseniia Boxleitner, Robert N. Spengler, Valentina Alekseitseva, Temirlan Chargynov, Aida Abdykanova, Nuritdin Sayfuloev, Svetlana Shnaider

**Affiliations:** 1 Max Planck Institute of Geoanthropology, Domestication and Anthropogenic Evolution Research Group, Jena, Germany; 2 Institute of Archaeology and Ethnography of the Siberian Branch of the Russian Academy of Sciences, Novosibirsk, Russia; 3 Department of Earth and Environmental Systems, Indiana State University, Terre Haute, IN, USA; 4 Kyrgyz National University Named After J. Balasagyn, Bishkek, Kyrgyz Republic; 5 American University of Central Asia, Bishkek, Kyrgyzstan; 6 Institute of History, Archeology and Ethnography Named After A. Donisha NAST, Dushanbe, Tajikistan; 7 International Research Laboratory ZooStan – ArchaeoZoological Center for the Study of Central Asia – CNRS – Al-Farabi Kazakh National University, IRL, Almaty 2033, Kazakhstan

**Keywords:** Paleobiology, Archeology

## Abstract

Central Asia, located at the crossroads of Eurasia, played a crucial role in the prehistoric spread of cultivated plants. Archaeobotanical evidence from rockshelters in the Fergana foothills and the Pamir Mountains reveals exchange between lowland and highland zones. Radiocarbon dating shows that broomcorn millet reached the lowlands of northern Central Asia by the late third millennium BCE and foxtail millet by the early second millennium BCE. Walnut and pistachio remains from the Fergana Valley indicate nut foraging as early as 7,800 years ago. The high-altitude site of Kurteke demonstrates cultural and economic links with lowland areas through shared technologies, while its plant assemblage differs from contemporaneous Tien Shan sites, suggesting distinct subsistence strategies. This study identifies early cultivation and foraging patterns along mountain ecoclines and proposes likely routes of crop dispersal across high-elevation Inner Asia, helping fill major gaps in the chronology of prehistoric trans-Eurasian agricultural exchange.

## Highlights

• Cultivation of broomcorn millet, wheat, and barley in the montane zone by 2000 BCE
• Findings of walnut and pistachio nut shells indicate the foraging of nuts from 5700 BCE
• Identification of preferred fuel sources in lowland and high-elevated sites

## Graphical abstract

**Figure:**
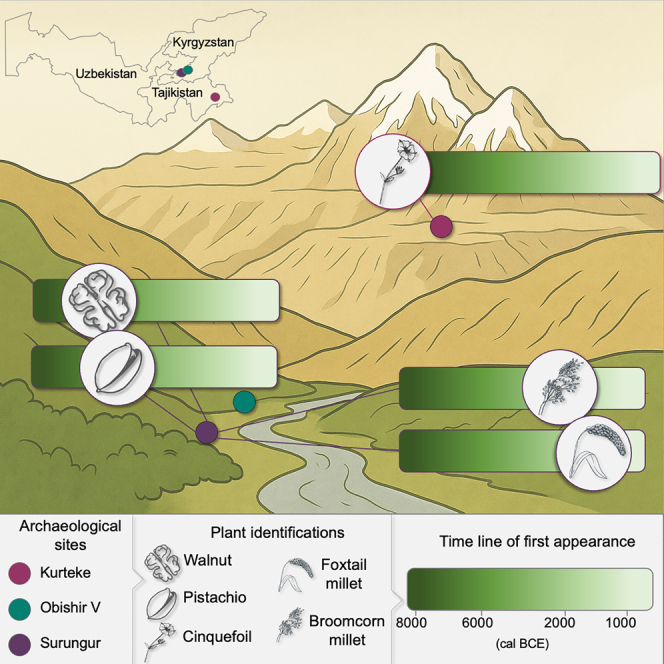

## Introduction

Caves and rockshelters have served as human refuges for millennia. The well-preserved sediments within these natural formations encapsulate detailed environmental, archaeological, and palaeontological data and often provide unparalleled archives of human activity. Investigating caves and shelters across diverse elevations and extensive chronological spans facilitates the reconstruction of dispersal routes of humans, as well as their associated fauna and flora; these data clarify the evolutionary transformations of these plants and animals and enhance our understanding of prehistoric social and cultural dynamics.

Cave excavations in Central Asia have consistently filled in gaps in the narrative of human prehistory. The Pamirs, Tian Shan, and Altai mountains formed a network of passes and river valleys serving as funnels for movements of populations of several hominin species in the Pleistocene.^1^^,^^2^^,^^3^ Illustrating the deep time depth of human presence in these caves, finds of Denisovans were first recovered at Denisova Cave in the Altai^4^ and later at Baishiya Karst Cave in the Qinghai Mountains of China.^5^^,^^6^ The cave of Teshik Tash in Uzbekistan revealed a Neanderthal presence in eastern Eurasia,^7^ with further possible occurrences of Neanderthal only at Kyrgyz Sel’Ungur, at Uzbek Anghilak, and at Tajik Khugji sites.^7^^,^^8^^,^^9^ Some caves were occupied for millennia, such as Tsagaan Agui Cave in Mongolia, where the analysis of stone tools and faunal remains has provided insights on the 500,000-year-long history of human populations in the region.^10^ While others sheltered humans for much shorter periods (e.g.,^11^^,^^12^^,^^13^^,^^14^^,^^15^^,^^16^^,^^17^^,^^18^).

**Figure 1:**
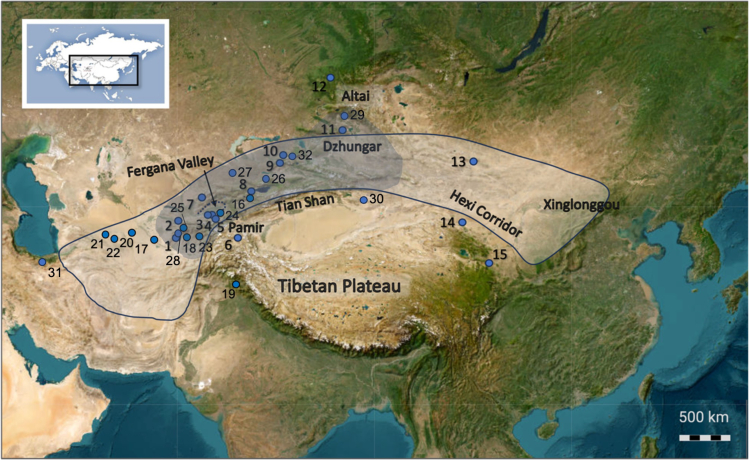
An overview map of the sites discussed in the paper: 1 Teshik Tash; 2. Sarazm; 3. Surungur; 4. Obishir V; 5. Chegirtke Cave; 6. Kurteke; 7. Obi Rakhmat; 8. Chap I and Chap II; 9. Begash; 10. Dali and Tasbas; 11. Tongtian Cave; 12. Denisova Cave; 13. Tsagaan Agui Cave; 14. Donghuishan; 15. Baishiya Karst Cave; 16 Aigyrzhal-3; 17. Adji Kui; 18. Kaynar Kamar; 19. Pethpuran Teng; 20. Gonur Depe and Togolok; 21. Anau; 22. Monjukli Depe; 23. Khugij; 24. Sel’Ungur; 25. Anghilak; 26. Kyzyl-Bulak I; 27. Ol-Dzhallau VII; 28. Toda Cave; 29. Ayituohan Ι; 30. Xiaohe; 31. Ghal e-Ben; and 32. Adungiaolu. The Inner Asian Mountain Corridor (IAMC) is highlighted in gray, while a northern route between Central Asia and China is outlined and highlighted in light gray. The background map is from ESRI World Imagery (nakarte.me).

The topography of Central Asia creates a complex system of microenvironments, with altitudinal zonality spread over 7,000 m, creating numerous biomes and ecotones. This variety of accessible microenvironments facilitated the spread of humans, who adapted subsistence practices to available local resources across Eurasia throughout the Holocene. Thus, easily accessible lowland regions and river valleys were the first to foster human movements across the region. The prehistoric diffusion of goods, ideas, and technologies through this mountain system into or from the oases of Xinjiang and the Hexi Corridor has been heavily discussed,^19^^,^^20^^,^^21^ gaining prominence after Frachetti^22^ coined the term Inner Asian Mountain Corridor (IAMC). In particular, the Fergana Valley is a natural corridor linking the Mirzacho’l Steppe to the Tian Shan and further north to the Dzhungar Mountains, which was crucial for promoting latitudinal exchange between East and West Asia^23^^,^^24^^,^^25^^,^^26^^,^^27^ (Figure 1). The role that Fergana and other river valleys play in the dissemination of agricultural crops and herd animals has only been systematically explored for the last decade.^23^^,^^28^^,^^29^^,^^30^^,^^31^^,^^32^^,^^33^^,^^34^

Over the past few years, scholars have turned greater attention to the cultural adaptations that permitted early humans to settle in the extreme landscapes of mountainous Inner Asia.^12^^,^^35^^,^^36^^,^^37^^,^^38^^,^^39^ The interest in high-elevation (2,500 m asl and above after Pawson and Jest^40^) adaptations has focused on the Himalaya, where scholars have proposed several competing (or complementary) cultural strategies for permanent human settlement. The first one to be discussed is a greater reliance on millet farming by 3200 BCE.^41^^,^^42^^,^^43^^,^^44^ Another proposed adaptive strategy is an implementation of a mixed millet-barley cultivation system developing from the second millennium BCE, with the consequent establishment of a barley-based economy.^45^^,^^46^^,^^47^ Other authors emphasize a crucial role of specialized hunting and fishing economies.^36^^,^^48^

**Figure 2:**
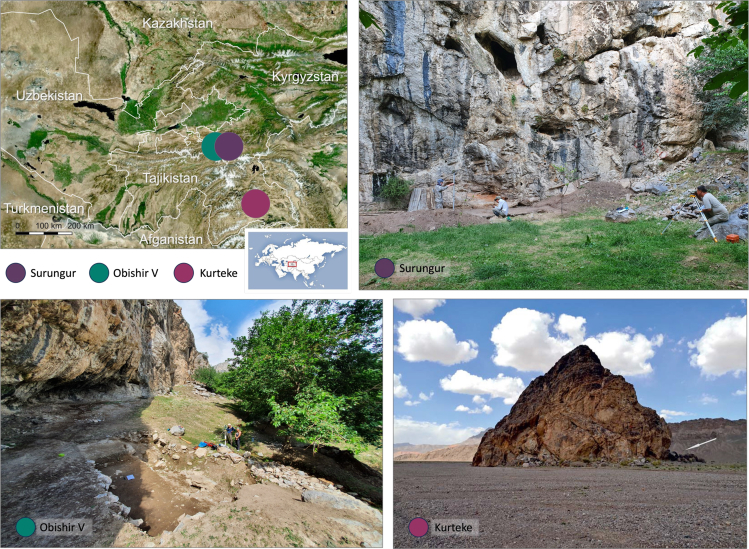
An overview map of the study region with a close-up of three archaeological sites sampled during the field seasons of 2018–2022 Photo of the Kurteke site is from Shnaider,^17^ photos of Surungur and Obishir V sites are from Boxleitner. A regional map from Planet.Nightly 210906.

Despite the rapid growth in archaeobotanical investigations in Central Asia,^23^^,^^29^^,^^35^^,^^49^^,^^50^^,^^51^^,^^52^^,^^53^^,^^54^^,^^55^^,^^56^ there are still lacunae in the understanding of the spread of early agricultural crops and cultivation practices within and across the region. The natural geographic gradient from lowlands to high mountains found in southern Kyrgyzstan and Tajikistan represents a suitable study area for understanding early foraging practices and the adoption of farming fostered by *trans*-altitudinal exchange in the region. Many cave sites and rockshelters (also referred to in the manuscript as “site”) located there were first described by Soviet scholars.^57^^,^^58^^,^^59^^,^^60^^,^^61^^,^^62^^,^^63^^,^^64^^,^^65^^,^^66^^,^^67^^,^^68^^,^^69^ A decade ago, some of these sites were revisited, and many more were discovered by PaleoCentralAsia, an international multidisciplinary team.^13^^,^^14^^,^^15^^,^^16^^,^^17^^,^^18^^,^^70^^,^^71^^,^^72^ Test pits and systematic excavations at the sites provide material essential for identifying human and animal diets, tracing domestication and cultivation, exploring trading and exchange routes, as well as reconstructing local vegetation communities during different stages of occupation. In this study, we focus on two archaeological sites in the southern Fergana Valley, Kyrgyzstan – the Surungur and Obishir V rockshelters – and one site in the Pamir Mountains of Tajikistan – the Kurteke Rockshelter. These three sites were sampled for archaeobotanical analysis during the field seasons of 2018–2022 (Figure 2, Table 1). The research aims of the current study are: to provide an overview of the plant assemblages utilized at the sites and their surroundings, to track the temporal spread of cultivated plants from lowlands to highlands, to derive information on paleodiets of humans and animals, and to gain insights into the practices of site usage.

### Archaeological sites and cultural contexts

#### Obishir V

Obishir V Rockshelter, located in the southern Fergana Valley of Kyrgyzstan (39°57′23.3″ N, 71°16′52.4'' E), was first studied under the direction of Islamov in the 1960s and 1970s. Based on the analyzed lithic artifacts and bones from the sediments, the original excavation team concluded that human occupation of the site started around 11,000–9,000 BCE.^73^ The cave became the eponymous site for the Mesolithic Obishirian Culture.^1^^,^^73^ Excavations at the site were renewed in 2015, identifying five stratigraphic layers.^13^ The upper stratigraphic layer (layer 1) dates from 2499 cal BCE to the present.^15^^,^^74^^,^^75^ Human presence is marked by finds of ceramic sherds, bone tools, modified bones of *Ovis/Capra* and other domesticated animals, e.g., cow and horse.^15^ The middle stratigraphic units (layers 2 and 3) cover the range of 7800-2500 cal BCE.^13^^,^^70^ Here, several types of stone tools characteristic of the Obishirian lithic industry, as well as grinding stones, were found, possibly suggesting the processing of plant material. Another prominent find from this part of the sequence includes bones of *Ovis* and *Capra* with traces of human modifications.^74^ A multidisciplinary analysis including ancient DNA, cementum analysis, and collagen fingerprinting together with the direct dating of the bones suggests an early dispersal of domestic sheep into the Fergana Valley by at least 6000 BCE, pushing back the accepted chronology of Neolithization of the region by 2,500 years.^74^ The lower unit of the site (layers 4 and 5) contained Epipaleolithic lithic assemblages of ca. 10,000-7000 BCE.^13^^,^^76^

Zooarchaeological studies^77^ show that much of the early Holocene faunal remains found at Obishir V represent rodents, lagomorphs, and bats. The dominant species in the paleofaunal assemblage was the Zaisan mole vole (*Ellobius tancrei*). The general composition of paleofauna coincides with modern animal communities of cave ecosystems. A constantly dry-warm climate and the establishment of steppe-like vegetation during the period of site formation are attested based on the analysis of fossil mollusks.^76^ The local ecology in the lower foothill zone would have been dry shrubby vegetation, with riparian forests along streams. Modern vegetation in this region is comprised of dry steppe communities at lower elevations and shiblyak (xerophylic) shrub floral communities with *Juniperus* forests from around 2,500 m asl and above.^78^ Wild fruit and nut forests are prominent, with *Juglans regia*, *Malus* spp., *Amygdalus communis*, *Pistacia vera*, *Prunus* spp., and *Morus nigra*, *Malus* spp., *Prunus* spp. growing in the vicinity of the site.

#### Surungur

The Surungur Rockshelter is located 2.5 km westward of Obishir V (39°57′23.8428″ N, 71°15′27.9432″ E). The site was discovered by Kasymov in 1972, but was not reported. Site was re-discovered in 2017 by the PaleoCentralAsia team and studied during the field seasons of 2018–2019 and 2021–2022 by the Central Asian Paleolithic Unit from the IAET SB RAS and the Balasagyn National Kyrgyz University. The results of a geophysical survey, including electrical resistivity tomography and magnetometry, suggest the presence of post-depositional disturbance in the upper part of the section, which might indicate traces of earlier unreported excavations. The lower part remained in an undisturbed state and, therefore, was promising for archaeological exploration.^79^ According to Olenchenko^79^ and Shnaider,^18^ there is solid archaeological evidence supporting human presence at the site over the past ten millennia. The upper stratigraphic layer (layer 1) is dated to 1410-500 cal BCE and contains eight hearths, burnt bone fragments, and shreds of ceramics attributed to the Chust Culture.^18^ Layer 2 covers a time range from 2500 cal BCE to about 5500 cal BCE. Human presence is marked by hearths, stone tools, and modified animal bones. In layer 3, several hearths and burnt stones were found; however, no direct dates are available for this stratigraphic unit. Macrocharcoal analysis of the Surungur deposits indicates that wood, bone, and dung were all used as fuel at the site, probably reflecting changes in fuel resource availability.^80^^,^^81^^,^^82^ Palynological analyses of five samples from Surungur revealed vegetation patterns and climatic conditions for each of three layers.^18^ Pollen spectra from layer 1 (ca. 500 cal BCE) with a high percentage of *Betula* spp. and *Salix* spp. grains; Amaranthaceae, Poaceae, and Asteraceae are interpreted as indicators of warm and humid climate. Three pollen samples from layer 2 suggest a shift from humid conditions identified based on the presence of *Juglans* sp., *Betula* sp., *Salix* sp., and *Artemisia* sp., Amaranthaceae, Cichorioideae pollen at ca. 4000 cal BCE to dry conditions with *Artemisia*, Amaranthaceae, Bignoniaceae, Plumbaginaceae, and *Ephedra* sp. at ca. 7500 cal BCE. Layer 3 provided pollen spectra interpreted as an indicator of cold and humid conditions.^18^ Modern vegetation around the site is similar to the plant communities around Obishir V, with dominant taxa of *Juglans regia*, *Malus* spp., *Prunus* spp., and *Elaeagnus angustifolia* cultivated around the site.

#### Kurteke

The Kurteke site is a 12 m wide, 3.5 m deep rockshelter, located at 3,980 m asl and approximately 40 km southeast of Murgab in the Eastern Pamir, Tajikistan (37°59′7.7964″ N, 74°25′53.8104″ E). This site preserves at least two cultural layers, which contain lithic artifacts, ceramics, and hearths attributed to the period ranging from the late 5th millennium to the end of the 2nd millennium BCE.^62^ In 2018, a Russian-Tajik expedition conducted palynological and zooarchaeological analyses and produced several direct radiocarbon dates on samples taken from a 0.8 m-deep test pit. Zhilich^83^ reports that the upper section of the pit (layer 1) is dated to ca. 3350-1550 cal BCE and contains pollen typical for dry *Artemisia* steppes and xerophilic plant communities. Zooarchaeological analysis identified an Equid tooth and *Ovis/Capra* tooth, and bone fragments belonging to sheep (*Ovis* sp.), bovid (likely sheep/yak), and “ungulate” (Cervidae/gazelle/saiga).^17^ Layer 2, dated to 11,783-11,287 cal BCE, contained pollen grains characteristic of a dry *Artemisia* steppe with a significant presence of coprophilic fungal spores and *Glomus*, suggesting herbivore activity.^83^^,^^84^ Faunal remains from this layer include *Ovis* and Leporidae.^17^^,^^84^ Evidence of human occupation is supported by the presence of hearths, burnt bones, and lithic artifacts. The stone tool assemblage bears similarities to the Obishirian cultural complex (ca. 7500-3000 BCE) from the Fergana Valley.^13^ Paleoecological data based on a pollen analysis of Lake Karakul, eastern Pamir, reveal a shift in floral composition in the mid Holocene.^85^ A dry, rather open, xerophytic vegetation was reconstructed for 10,950–4750 cal BCE. During the next analyzed period, 3450 cal BCE – 950 cal CE, a shift to an increased proportion of steppe and meadow-steppe taxa was suggested to reflect an increase in moisture availability.^85^

Kurteke is located on the high plateau of the eastern Pamir with an annual precipitation of 70–120 mm.^86^ The plateau consists of semi-arid deserts and mountane deserts (3500–4600 m al), vegetation of subalpine (3500–4200 m al), cryophilus steppes and alpine meadows (4100–4800 m asl), and sparse nival vegetation (above 4700 m asl). Saline marshes and shores of lakes and ponds are mainly occupied by Poaceae, Asteraceae, Amaranthaceae, and *Polygonum sibiricum* var. *thomsonii*^87^*.* Cryoxerophytic vegetation of high elevations is dominated by Amaranthaceae, Asteraceae, Brassicaceae, Poaceae, and Rosaceae.^88^

## Results

### Chronology

**Figure 3:**
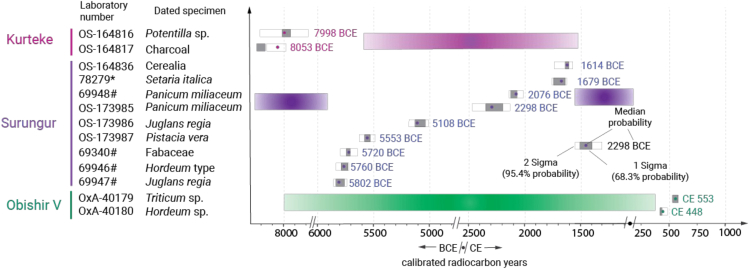
Results of the direct radiocarbon dating of plant macrofossils from Obishir V, Surungur, and Kurteke OS- marks Woods Hole radiocarbon lab, octothorpe indicates CAIS lab, OxA stands for the Oxford Radiocarbon laboratory, asterisk marks the Curt-Engelhorn-Center Archaeometry GmbH, Mannheim, Germany. Colored blocks indicate the chronological time span covered by the sediments of the corresponding site, but not directly dated by the current project.

Sedimentation, accumulation, and associated processes within the rock shelters may pose difficulties in interpreting the archaeological data. Thirteen charred plant macrofossils were directly dated to cross-check the chronology of site units and avoid discrepancies in interpretations. A wheat grain from layer 1 of Obishir V was dated to 528–579 cal CE, and a barley specimen from the same layer to 415–485 cal CE. Nine plant remains from Surungur Rockshelter provide the following chronological frame: ca. 1600–2300 cal BCE for layer 1; 2076–5500 cal BCE for layer 2. Three samples from layer 3 reported a range of 5700–5800 cal BCE. Two charred plant remains from Kurteke indicate site occupation during 7998–8053 cal BCE. An overview of the results is available in Figure 3, detailed information is provided in Table S1.

### Plant macrofossils

#### Obishir V

During the field seasons of 2018, 2019, and 2021, a total of 80.65 L of sediment was floated from Obishir V. The flotation yielded 1,546 seeds and large seed fragments across 13 samples (Table S2), along with 157 egg shell fragments and 48 small bone fragments. Additionally, 15,176 charcoal fragments larger than 2 mm were recovered. The majority of the identified plant remains (*n* = 928) were seeds from the Amaranthaceae family.

**Figure 4:**
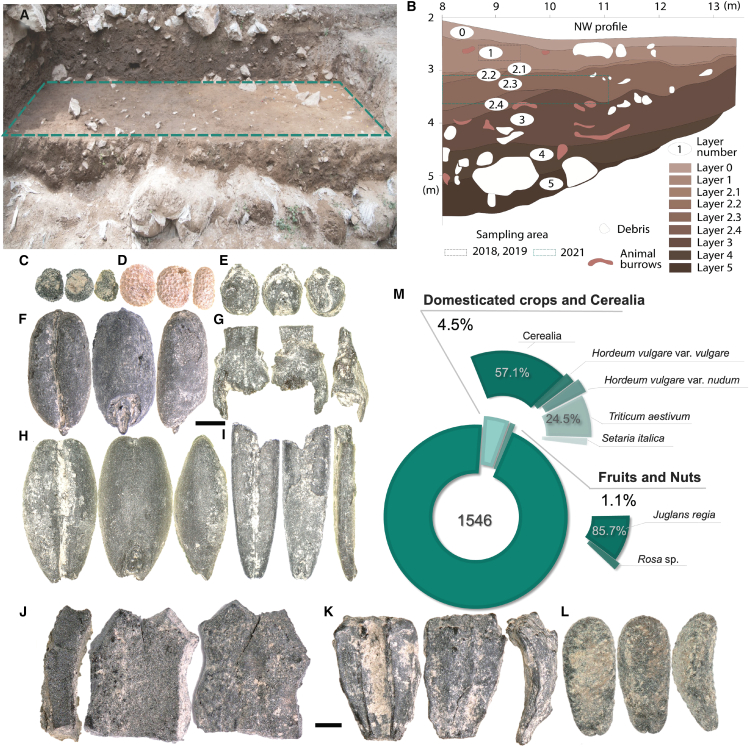
Overview of the Obishir V site (A) sampled surface with layers 2.2–3; (B) a scheme of stratigraphic layers of the studied area (modified after Brancaleoni^71^) with a sampling area of 2021 outlined with green dashed line; (C-L) plant remains recovered at the site: (C) Caryophyllaceae; (D) *Hyoscyamus niger*; (E) Foxtail millet (*Setaria italica*); (F) *Triticum aestivum*; (G) *Triticum aestivum* rachis; (H) naked barley (*Hordeum vulgare* var. *nudum*); (I) *Bromus* sp.; (J) walnut (*Juglans regia*); (K) *Xanthium strumarium*, (L) *Onopordum acanthium*; (M) pie chart featuring plant assemblage recovered at the site. Black bar indicates 1 mm; note different magnifications for C-I and J-L plant remains.

The 2018 sample originated from layer 1, dated to 2499 cal BCE to the present.^15^^,^^74^^,^^75^ It contained a diverse array of carbonized plant remains, including both domesticated crops (6.3%) and wild plants together with fruits and nuts (93.7%). We identified 21 domesticated grains and grain fragments, indicating both naked and hulled barley (*Hordeum vulgare* var. *nudum* and *H. vulgare* var. *vulgare*, respectively), free-threshing hexaploid wheat (*Triticum aesitivum*), and two foxtail millet grains (*Setaria italica*) (Figure 4). Additionally 48 grain parts were found, including barley and wheat rachises, indicating that both barley and wheat were present or processed at the site. Other finds from layer 1 included eight walnut shell fragments (*Juglans regia*) and two seeds of *Rosa* sp. Wild seeds composed 93.8% of the sample. The most common wild seeds recovered were Amaranthaceae. Other identified wild taxa include five types of Poaceae, two types of Fabaceae, several Asteraceae (e.g., *Xanthium strumarium* and *Onopordum acanthium*), Convolvulaceae, *Potentilla* sp., Polygonaceae, *Malva* sp., *Galium* sp., *Thymelaea* sp., *Polygonum* sp., Solanaceae, Lamiaceae, Boraginaceae, Brassicaceae, and a twig of *Juniperus* sp.

No domesticated grains or plant remains of cultivated species were found in the 2019 and 2021 samples, which correspond to layers 2 and 3, dated to 7800-2500 BCE.^13^ The wild taxa included Amaranthaceae, Fabaceae, *Lithospermum arvense*, *Euclidium syriacum*, *Trigonella* sp., *Potentilla/Fragaria* type, and showed a lower abundance of identified macrofossils than were found in layer 1. However, three walnut shells were identified in layer 2. Notably, the 2021 samples, from layers 2 and 3, contained eggshell fragments (*n* = 157), which were absent from the layer above.

#### Surungur

**Figure 5:**
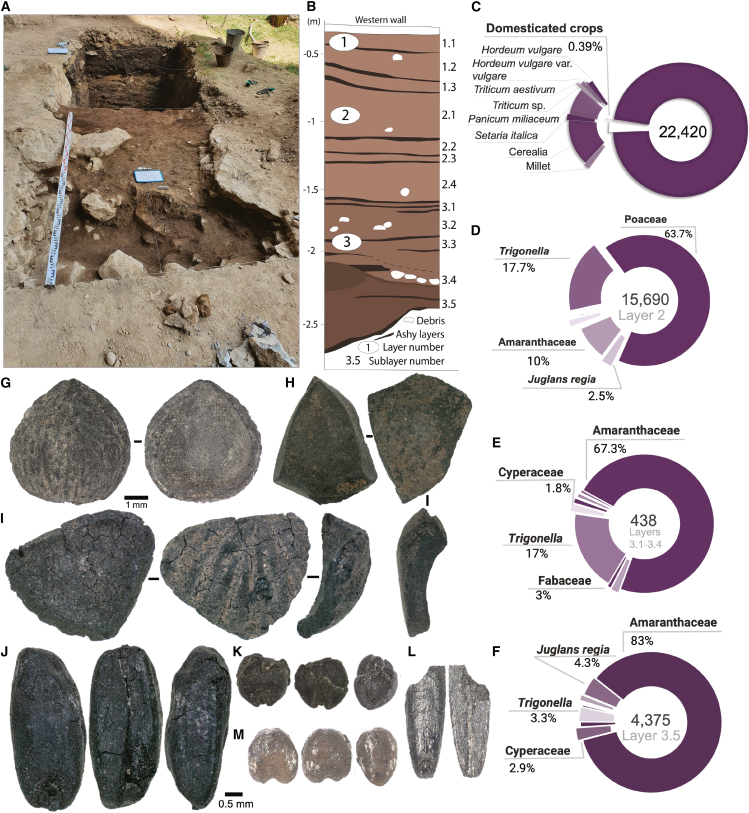
Overview of the Surungur site (A) sampled surface with layer 2.2; (B) a scheme of stratigraphic layers of the studied area (modified after Shnaider^18^). Pie charts feature plant assemblages recovered at the site (C) and in layers 2 (D), 3 (E), and 3.5 (F). (G-L) plant remains recovered at the site: (G) *Prunus verrucosa*; (H) shell fragment of walnut; (I) shell fragment of pistachio; (J) barley (*Hordeum vulgare*); (K) foxtail millet (*Setaria italica*); (L) *Bromus* sp.; (M) broomcorn millet (*Panicum miliaceum*). Black scale indicates 0.5 mm; note a separate scale for (g).

Layers 1, 2, and 3 of Surungur were studied during the field seasons of 2019, 2021, and 2022. As a result, 92 samples were collected using a blanket sampling strategy for archaeobotanical analysis. A total of 897.95 L of sediment were floated, leading to the recovery of 22,399 plant seeds and fragments (see Table S3). In addition to identifiable plant remains, we also found charcoal (>2 mm, *n* = 19,742), small bones and bone fragments (*n* = 593), egg shells (*n* = 126), and snails (*n* = 362). Ten of the samples yielded no macrofossil remains other than charcoal and are therefore reported separately in Table S4. The majority of the plant remains consist of wild seeds (*n* = 21,132). The most abundant remains across the studied layers include Poaceae (*n* = 10,023), Amaranthaceae (*n* = 7,319), and *Trigonella* sp. (*n* = 3,040). In addition, we identified several other wild plant taxa, including three types of Fabaceae (besides *Trigonella* sp.), species from the Brassicaceae, Caryophyllaceae, Polygonaceae, Asteraceae, Convolvulaceae, and Lamiaceae families, as well as *Capparis spinosa*, *Hyoscyamus niger*, *Potentilla* sp., *Malva* sp., *Galium* sp., and *Thymelaea passerina* (Table S3). Fragments of nut shells and fruit stones totaled 722. Identified fragments belong to walnut (*n* = 604), pistachio (*n* = 2), hackberry (*Celtis caucasica*) (*n* = 4), and *Prunus verrucosa* (*n* = 2) (Figure 5).

Among the seeds recovered from Surungur, we found 36 domesticated grains and grain fragments. These include both broomcorn and foxtail millets (*Panicum miliaceum* and *Setaria italica*, respectively), hulled barley (*Hordeum vulgare* var. *vulgare*), and free-threshing hexaploid wheat (*Triticum aestivum*). Despite the fact that most of the recovered plant taxa were present in all layers, the share of domesticated grains, fruits, and nuts, and wild taxa slightly differs across the layers. The plant assemblage of layer 2 comprises 15,690 plant remains, and the share of the wild plants (99.6% of the total plant remains recovered in layer 2) is dominated by Poaceae (63.7% of all identified remains), followed by *Trigonella* sp. (17.7%), Amaranthaceae (10%), and *Juglans regia* (2.5%). Only this layer yielded the presence of *Prunus verrucosa* and *Pistacia vera*. We identified 33 (0.4% of the total count for the layer 2) domesticated grains, including *Panicum miliaceum, Setaria italica*, *Hordeum vulgare* var. *vulgare*, and *Triticum aestivum*, as well as two wheat rachises in the samples from layer 2. The presence of rachises provides evidence that hexaploid wheat was either present or processed at the site. Layers 3.1–3.4 provided 438 plant remains for archaeobotanical studies, with 99.7% being identified as wild plants and nuts and 0.3% attributed to domesticated grains. The most abundant taxa belonged to Amaranthaceae (67.3%), *Trigonella* sp. (17%), and Fabaceae (3%). Only three unidentifiable nut shell fragments and no domesticated grains were found here. The lowermost sublayer 3.5 yielded 4,375 seeds and seed fragments, with Amaranthaceae (83%) being the most abundant, followed by *Trigonella* sp. (3.3%), Cyperaceae (2.9%), and *Juglans regia* (4.3%). We recovered 15 fragments of cereal grains, some of which were attributed to barley.

#### Kurteke

**Figure 6:**
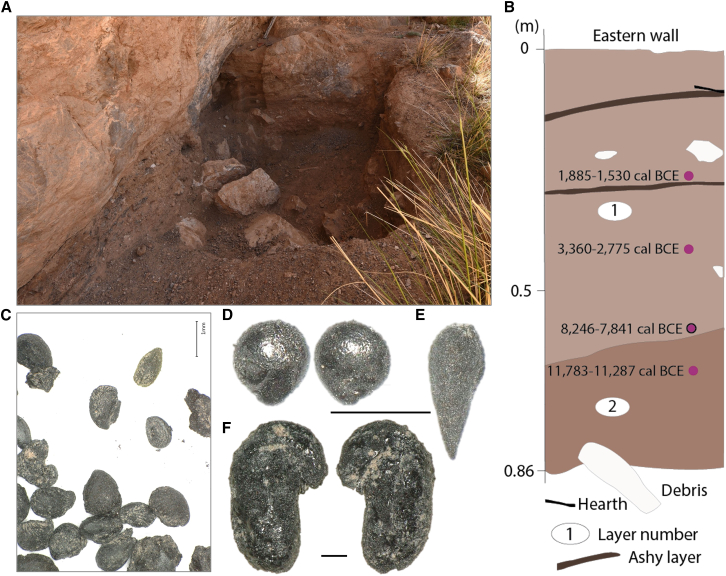
Kurteke site (A) excavation area (photo by Shnaider); (B) stratigraphy of the test pit layers, black stroke indicates where analyzed sample was taken (modified after Shnaider^84^); plant remains recovered at the site (modified after Shnaider^84^): (C) *Potentilla* sp., (D) *Chenopodium iljinii*, (E) *Thymelaea passerina*, (F) Fabaceae. Black scale indicates 1 mm; note a different magnification for the Fabaceae seed.

A total of 382 seeds and seed fragments were recovered from the 4-L sample. In addition to the carpological material, 1,292 carbonized wood fragments and 214 small bones were found (Table S2). The plant assemblage consists exclusively of wild seeds, with Rosaceae comprising 65% of them. Notably, some of the *Potentilla* spp. seeds are preserved in dung (Figure 6); however, precise identification of the plant to the species level was not possible due to preservation conditions. According to Nowak and Nobis,^87^ five species of *Potentilla* grow in the region: *Potentilla agrimonioides*, *P. anserina, P. arnavatensis, P. pamiroalaica,* and *P. tephroleuca*, and Kurteke assamblage might include multiple of the autochthonous species. Other identified plant taxa include cf. *Chenopodium iljinii*, *Carex stenophylla* sp. *stenophylloides*, Poaceae, *Thymelaea passerina*, and Fabaceae. While 27 species of Fabaceae grow in the region,^87^ precise identification of the Fabaceae remains was not possible due to the absence of reference collections and the high number of wild species.

## Discussion

While we provide new insights into the dynamic spread of crops, domesticated animals, and technologies in the Bronze Age (3300-1000 BCE) along the IAMC, the data for this broad ecoregion are still limited. The scholarship shows eastward movement of wheat, barley, sheep, and goat^23^^,^^34^^,^^89^ and westward movement of millets^90^ and references therein. Wheat appears at Tasbas I, eastern Kazakhstan, at 2840–2490 BCE,^23^ and arrives in China between 2200 and 1800 BCE.^90^ It took a millennium for barley to travel along the IAMC from Sarazm, Tajikistan (third millennium BCE^91^) to Donghuishan, China (1608-1434 BCE^89^). Appearance of domesticated sheep, identified at Obishir V, Kyrgyzstan, is dated to the 6000 BCE^74^; while domesticated sheep and goat reached China by 2000-1900 BCE.^90^ Westward movement of millets (broomcorn and foxtail) from Hebei, northeastern China, where they evolved domestication traits by 6500-5500 BCE,^90^ through the IAMC is also attested to the Bronze Age. Broomcorn millet, together with wheat and barley, was recovered at Begash, Kazakhstan, at 2200 BCE,^34^ while foxtail millet arrived by c. 1400 BCE at Tasbas, Kazakhstan.^23^^,^^32^

While the foothills clearly attracted human attention throughout the Holocene, the higher elevation ecotones represent a more formidable landscape. Recent ethnographic work in the montane areas describes several models of high-elevation resource use, including practicing progressive harvesting, hunting using vantage points, direct access to sources for metallurgy and rock utilization, and accessing seasonal pastures.^92^^,^^93^^,^^94^ This inevitably leads to the questioning of the degree of mobility within the pastoralist system. Archaeologists in Central Asia for decades have echoed ethnographic observations that the adoption of diverse subsistence strategies (multi-resource, vertical transhumant, agropastoral) with a varying level of mobility (sedentary, seasonal migration, year-round mobile) co-existing in the high-elevation areas enabled the exploration and settlement of these areas.^6^^,^^12^^,^^29^^,^^36^^,^^44^^,^^47^^,^^95^^,^^96^^,^^97^^,^^98^ Illustrating the diversity in economic practices, archaeological studies at Tongtian Cave in the Altai Mountains, unearthed cultural links between East and West Asia and show evidence of spread of wheat, barley, and sheep, into the northern Asian regions starting from the fourth millennium BCE.^12^ Evidence from ca. 2200 BCE for an agropastoral subsistence based on breeding sheep at 3,000 m asl comes from the Chegirtke Cave, Kyrgyzstan.^11^ Recent discoveries at Obishir V, Kyrgyzstan, have pushed back the dates for the appearance of the first domesticated animals (sheep and goat) in Central Asia to the sixth millennium BCE.^74^ A multi-crop cultivation system was identified at Chap II (2,000 m asl), Kyrgyzstan consisting of free-threshing wheat, glume wheat, and hulled and naked barley, along with sheep and goat, and dates to third millennium BCE.^31^ The results of archaeobotanical investigations from the Fergana Foothills and the Pamirs are adding crucial information about agricultural practices on the western edge of the IAMC.

### Wild plant seeds and their sources

The majority of identified plant remains at the discussed sites belong to weedy wild plants, which reflect local environments. In the lowlands, at Obishir V and Surungur, the most abundant botanical remains were Amaranthaceae, Fabaceae, and Poaceae seeds. While at the high-elevation site of Kurteke, the majority of identified seeds were *Potentilla* sp. Short-growing woody species of *Potentilla* are prominent at higher elevations in the montane regions of Eurasia and serve as a food source for grazing animals.^99^^,^^100^ Modern observations of grazing pressure produced by large herbivores on vegetation in the Eurasian steppes also suggest that *Potentilla* species are abundant on heavily grazed landscapes.^101^ Both regions are characterized by a highly continental climate and, therefore, invite comparisons. Dung burning provides a likely vector for the carbonized seeds of these plants to enter the anthropogenic sediments. Herd animal dung would have been an especially important fuel source at higher elevations where tree species are absent or scarce. In the lowlands, a detailed investigation of the burned horizons at Surungur, together with a palynological analysis of the sediments, suggests that dung, bone, and wood were all used as fuel.^80^ These data support the assumption that macroremains might indicate dung burning practice, as reported for many other archaeological sites in Central Asia (e.g., Spengler^102^).

**Figure 7:**
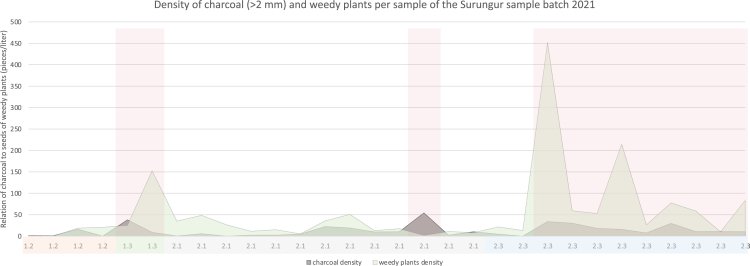
A graph illustrates the distribution of charcoal and weedy seed density in the samples from Surungur (field season 2021) Red areas indicate samples taken from fireplaces and hearths.

**Figure 8:**
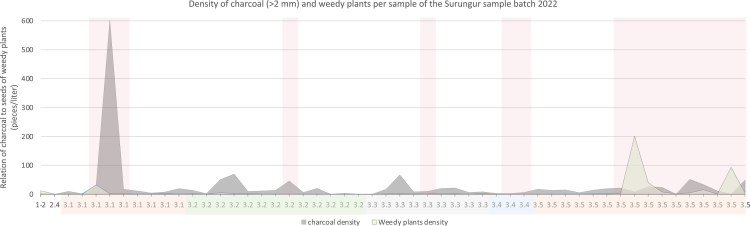
A graph illustrates the distribution of charcoal and weedy seed density in the samples from Surungur (field season 2022) Red areas indicate samples taken from fireplaces and hearths.

In order to test the theory that a dung-burning signal is visible in the archaeobotanical assemblage at Surungur, we plotted the data for abundance of weedy seeds against charcoal fragments (Figures 7 and 8) and compared the observations with the analysis of the burnt horizons made by Dedov.^80^ According to macrobotanical analysis, layers 1 and 2 are characterized by an abundance of weedy remains. However, a closer look at the samples originating from fireplaces and hearths reveals peaks in concentrations of charcoal and weedy plant remains (Figure 7). High densities of both charcoal and weedy seeds were found in two hearths from sublayer 1.3, while only one fireplace in sublayer 2.1 was characterized by the abundance of charcoal, and nine further fireplaces from sublayer 2.3 revealed the prevalence of herbaceous plant remains. These imply that both wood and dung were used as fuel sources in parallel during the middle and late Holocene. Such assumptions on fuel choices based on archaeobotanical analysis are in agreement with the previous investigations of sediments at the site, which identify that a mix of grasses, dung, and wood was utilized as fuel.^80^^,^^82^ Layers 3–3.4 are characterized by a prevalence of charcoal remains over weedy remains with clear concentration peaks at ashy spots and fireplaces. However, apparent peaks in weedy plant density curves are registered in three fireplaces of sublayer 3.5, suggesting that dung could have also been used as a fuel source during the temporal use of the site. These observations are in line with the assumption by Dedov,^80^ stating that during the formation of this layer, wood and grass were mainly used as fuel.

At the Kurteke site, the scarcity of tools and bones,^17^ together with the low abundance of plant remains recovered from the only fireplace found, suggests that the site was used sporadically. The plant assemblage from Kurteke exclusively contains the remains of weeds. Numerous species of Fabaceae, Poaceae, and *Chenopodium* in the high-elevation region of the southeastern Pamir are plants that can be foraged by both herbivores and humans.^87^ Ethnographic observations of high-elevation Tibetan communities^103^ suggest that a diverse array of plants have been used for food in extreme environmental conditions and implies that “herding in the Pamirs is a combination of vertical transhumance and semi-nomadism.”^104^ Summer pastures are located at elevations between 3,500 and 4,700 m asl, and winter pastures are at lower elevations.^104^ A similar pattern of herding or following the seasonal migrating herbivores would explain the exchange of commodities and a resulting common lithic tool industry present at both high-elevation Kurteke and low-elevation sites of Obishir V and Obishir I, dating back at least to 8,000–6,000 years ago.^13^

### Walnuts and pistachios

Nut shells were recovered only at the Fergana Valley sites of Obishir V and Surungur, hinting at the likelihood that people were foraging locally available resources as opposed to carrying them to consume elsewhere. The walnut shells (*Juglans regia*; Surungur, *n* = 604; Obishir V, *n* = 11) are significant, as they indicate a deep human connection with this species in Central Asia. At Obishir V, the presence of stone percussive-abrasive tools (*n* = 3), including two grinding plates and a pestle-anvil tool, suggests plant processing activities at the site.^105^ These tools might have been used for processing walnut and pistachio nuts that grew in the vicinity of the site. The native range of walnuts is often ascribed to a foothill belt spanning southwestern Kyrgyzstan, Uzbekistan, and Tibet, along with northern India and Pakistan, Turkmenistan, Iran, and the Caucasus^106^ and references therein. Cultivation of walnuts likely originated across its wide range.^107^^,^^108^^,^^109^ Active and intensive management of walnuts by humans is confirmed only for the last 2,400 years, notably across the Roman Empire (e.g.,^110^^,^^111^^,^^112^^,^^113^^,^^114^). In Central Asia, walnuts are assumed to have been cultivated or maintained only by the second half of the first millennium CE.^51^ Palynological data from Beer et al.^115^ suggest that walnut forests were absent along the slopes of the northern Fergana Valley prior to 50 BCE. However, finds of walnut pollen grains from within six lake’s sediments dated to ca. 4000 BCE are extremely rare, which has been interpreted as suggesting that these pollen grains had dispersed from a long distance away.^115^ The data from the current study might indicate a source of the few walnut pollen grains discussed by Beer et al.^115^ The traveling distance and prevailing wind direction across the valley might have reduced the number of pollen grains reaching the lake sediments in northern Fergana. The results of at least one palynological investigation of Surungur layers (unpublished), together with our findings of walnut shells in layers 1–3 at the site, show that walnuts are native to the region and for the first time confirm the intentional usage of nuts from walnut trees at least during the last 7,800 years in the southern Fergana Foothills.

We found fragments of shells (endocarps) of pistachios in two samples from Surungur, directly dated to 5626-5481 cal BCE (Table S1). Archaeological evidence for the consumption of pistachios in Central Asia before the Common Era is scarce. The earliest recovered archaeobotanical remains of pistachio were found at the Kaynar Kamar Rockshelter, Uzbekistan, suggesting consumption by at least the ninth millennium BCE,^116^ followed by finds of pistachio endocarps at the Toda Cave, Uzbekistan, in the layers dated to the eighth millennium BCE.^117^ Pistachio remains were also found at Shortughai, northern Afghanistan,^118^ dating to the third millennium BCE, and at the second millennium BCE site of Sarazm, Tajikistan.^91^ Also, fragments of nut shells were identified in the Kaptar Kamar Cave^119^ and Djarkutan, Uzbekistan,^120^ within the layers dated to the second millennium BCE. Central Asia, including Kyrgyzstan, harbors wild pistachio stands and is one of the proposed centers of origin and domestication,^121^^,^^122^ with Syria and eastern Anatolia being suggested as an alternative center of origin.^123^^,^^124^ Current data suggest that it was brought under cultivation within the past 3,000 years in southern Central Asia and spread southwards and westwards by the end of 1000 BCE and eastwards by the end of the first millennium CE.^119^ Our finding of pistachio confirms that these nuts grew wild in the Fergana Valley and were consumed by humans for at least the last 7,500 years.

### Cultivated plants

At Obishir V, grains of domesticated crops, barley, wheat, and foxtail millet, were recovered from only one sample. The barley and wheat grains from this sample date to the 5-6^th^ centuries CE (Figure 3; Table S1). By the Early Middle Ages and prior to the Arab Conquest, complex agricultural practices were already being widely implemented across the broader region^50^; therefore, the presence of wheat, barley, and foxtail millet at Obishir V is not surprising.

At Surungur, finds of wheat, wheat rachises, barley, broomcorn and foxtail millets, and Cerealia grains in layers 1 and 2 of the site support the results of palynological analyses by Zhilich et al.^83^ and starch grain analysis of the stone tools (*n* = 6).^125^ Zhilich found pollen with Cerealia-type features in layer 2.2 and above, suggesting the intentional cultivation of those near the site, while starch grains preliminarily identified as millet, and taxa from the Fabaceae family were found on stone tool surfaces from layer 1. The results of starch analysis of the stone tools from layers 3 (*n* = 1) and 4 (*n* = 6) revealed the presence of barley type starch and those from the Liliaceae/Solanaceae families.^125^

Broomcorn millet was domesticated on the North China Plains and became a staple food there by 6500 BCE.^126^^,^^127^ By 3000-2500 BCE, it reached southeastern and southwestern China and Taiwan.^90^^,^^128^ However, the timing and the route of spread of broomcorn millet outside of China is still debated. For example, Karuo in southeastern Tibet provided finds of broomcorn millets dating to 2900–2600 BCE,^44^ along with millets from Baiyangcun site, Yunnan, China, dated to 2868-2573 BCE.^129^ Debate continues over the possibility of a southern route of millet spread around the Himalaya or north via the Hexi and Inner Asian Mountain corridors (see Table S5 for an overview of discussed millet finds). Finds of millets in northwestern China, at Tongtian Cave (2199-1981 cal BCE^12^) mark the northern limit of this crop in the third millennium BC. Frachetti et al.^34^ provided direct dates of 2460-2150 cal BCE on broomcorn millet grains that were recovered from the Begash site in southeastern Kazakhstan. Another noteworthy line of evidence comes from isotopic analysis of human remains, which attest to a C_4_ component in the diet, which was likely broomcorn millet. Early C_4_ signals come from the human individuals from the Ayituohan I Cementry, China, dated to 2836-2490 cal BCE,^130^ followed by isotopic signal suggesting reliance on the millet diet from the Dali site, Kazakhstan, for 2705-2545 cal BCE^131^ and the Aigyrzhal-3 site, Kyrgyzstan, for 2460-2204 cal BCE.^30^ Additionally, in Turkmenistan at the Togolok site, millets returned an age of 2197-1983 cal BCE,^52^ and a roughly contemporaneous date for broomcorn millet grains was reported from further southwest, at the Pethpuran Teng site in the Kashmir Valley, where 100 millet grains were dated to 2500-1950 BCE.^132^ Further finds of broomcorn millet are available at Adji Kui in Turkmenistan from the layers dated to 2200 BCE,^133^ at the Xiaohe site in China, where broomcorn millets were dated to 2011-1756 BCE,^89^ and millets discovered at Ghal e-Ben in northern Iran are dated to 2141–1951 BCE.^134^ The dates of the two broomcorn millet grains from Surungur are 2465-2131 cal BCE and 2143-2007 cal BCE, making them the second oldest along the IAMC, adding to the few directly dated millet remains found in Central Asia.

Foxtail millet originated from a similar domestication center as the broomcorn millet – in northern China^90^ – reaching southeastern Tibet, at the Karuo site by 2700-2300 BCE.^47^ Also from Dali, foxtail and broomcorn millets were identified in pottery impressions found within layers dated to 2850-1550 BCE.^135^ However, foxtail millet appears in the Central Asian archaeobotanical record by 1400 BCE at Tasbas, Kazakhstan,^23^^,^^32^ at 1366-1124 BCE at Uch-Kurbu, Kyrgyzstan (Motuzaite Matuzeviciute et al., 2022a), and at 1065-825 cal BCE at Chap I.^28^ Also, several foxtail millet finds appear right after 2000 BCE at Surkotada and Ojiyana in India,^136^^,^^137^ suggesting another possible route of foxtail millet spread via southern Tibet instead of the IAMC. Therefore, every new directly dated foxtail millet grain from the Bronze Age of Central Asia represents valuable evidence helping to pinpoint its trajectory of spread from China to Europe. Four grains of foxtail millet were found at Surungur. One of the grains originating from layer 2 was directly dated to 1741-1617 cal BCE.

Agriculture with free-threshing wheat and barley cultivation spread into Central Asia between the second half of the sixth and fifth millennia BCE as suggested by archaeobotanical studies of sediments from Jeitun,^138^ and Monjukli Depe in southern Turkmenistan.^139^ Both wheat and barley were recovered at the Chalcolithic Anau North site (4500-3000 BCE) from southern Turkmenistan. Archaeobotanical data from Sarazm,^91^ Tajikistan, confirm the spread of cultivation of wheat and barley to the northeast in the course of the fourth millennium BCE. Further evidence of barley and wheat cultivation from Bronze Age sites in Turkmenistan, notably Gonur and Anau South (3000-2700 BCE), and in southern Uzbekistan at Djarkutan,^120^ suggests that southern Central Asia was an agricultural region. However, the vast area to the north remained little explored in terms of the spread of cultivars. The earliest identified wheat grain in northern Central Asia has been recovered from Tongtian Cave, Altai Mountains, dated to 3098-2916 cal BCE.^12^ After a 400-year hiatus in the archaeobotanical record in northern Central Asia, wheat grains unearthed at Tasbas I, were dated to 2600 cal BCE,^140^ while wheat together with barley and broomcorn millet were found at Begash, dated to 2200 cal BCE.^34^

A fragment of a barley grain found at Surungur was directly dated to 5795-5641 cal BCE (Table S1). This single grain fragment is narrow and symmetrical and has a broad furrow, which is characteristic of a hulled barley. However, it is impossible to reconstruct the size of a complete grain and identify if the specimen belongs to a domesticated or a wild plant. There are six species of barley growing in the region today, and they could have been foraged in prehistory: *Hordeum bogdanii, H. brevisubulatum, H. bulbosum, H. marinum* ssp*. gussoneanum, H. murinum* ssp*. leporinum,* and *H. vulgare* ssp*. spontaneum*.^141^ Given what is preserved of the specimen, it looks morphologically wild, stressing that further archaeobotanical analyses are necessary for a better understanding of the utilization of barley in the region. The likelihood of wild grain harvesting is supported by evidence from another site further south in Central Asia. Archaeobotanical data from Toda Cave in southern Uzbekistan revealed insights into subsistence strategies of local populations starting from the tenth millennium BP.^117^ Among others, several grains of barley were recovered from the cave and directly dated to 7301-6829 cal BCE, 7039-6698 cal BCE, and 6075-5989 cal BCE.^117^ A detailed morphological comparison of these barley grains to available pre-domesticated and domesticated specimens from southwest Asia led to an assumption that the assemblage of the cave consists of a wild form of barley that was harvested in Central Asia, starting at least 9,000 years ago.^117^ Contemporaneous archaeobotanical material with layer 3.5 of Surungur was recovered at the Kaynar-Kamar site in southern Uzbekistan,^116^ but only remains of pistachio nut shells were found at the site.

Our archaeobotanical investigations at Surungur reveal wheat, barley, and cereal grains in the layer covering a time span starting from 2465 to 2131 cal BCE (direct date on a broomcorn millet grain) to 1741-1617 cal BCE (direct date on a foxtail millet grain). Findings of cultivated crops, especially grains with almost no by-products, may suggest that the crops were not grown at the site, but rather carried to the site. We are cautious regarding any discussions of the role of these grains in the overall economy, due to the limited number of finds; however, their presence suggests that people in the Fergana Valley were either tied into an exchange network or transporting grains with herd movements.

The archaeobotanical assemblages from Surungur, Obishir V, and Kurteke address questions of the foraging of wild nuts and fruits, the cultivation of grain crops, and fuel choices by people inhabiting the southern Fergana Valley and the eastern Pamirs. A grain of a morphologically wild form of barley found at Surungur and directly radiocarbon-dated to the sixth millennium BCE provides a valuable insight into Neolithic foraging practice in the Fergana Foothills, as this find contributes to the limited list of the earliest barley-type grains recovered in Central Asia. The presence of both broomcorn and foxtail millet in Bronze Age layers of Surungur expands the currently small dataset of the earliest appearance of millets along the IAMC. These data help to contextualize the westward spread of domesticated millet from its origins in China through Central Asia toward Europe. Direct dating of walnut and pistachio nut shells has confirmed that the wide range of these trees included the Fergana Valley, a previously disputed claim. These findings represent the oldest evidence of nuts in the region, suggesting that the foraging of nuts was practiced at 5700 BCE. We also identified preferred fuel sources by comparing the densities of weedy plants and charcoal in the samples and complementing the existing fuel studies at these sites. At lowland sites, both dung and wood were utilized for fuel, with distinct changes in preference corresponding to shifts in regional climatic conditions throughout the Holocene. At the high-elevation site, woody plants seemed to have been the preferred fuel source at around 8000 BCE.

These findings contribute to the limited archaeobotanical data available from Central Asia prior to the third millennium BCE. Alongside reported direct dates on plant remains, the evidence indicates a broader tradition of agropastoralism and crop cultivation, including broomcorn millet, wheat, and barley, in the montane zone by 2000 cal BCE. Further excavations of the sites with synchronous occupation spans in the foothills of the Central Asian mountain zone, and the high-elevation settings of Tian Shan and the Pamirs will enhance our understanding of the spread and timing of crop cultivation, domestication, and shifts in subsistence strategies in response to changing climatic conditions during the Holocene.

### Limitations of the study

In the current study, the volume and number of samples from the high-elevation Kurteke site were limited. Additional samples from the site could deepen our understanding of past subsistence strategies and provide further insights into site utilization patterns (e.g., seasonality and mobility). Anthracological analysis was not a part of the current study; however, it could have offered additional information on the use of plant resources at the studied sites.

## Resource availability

### Lead contact

Requests for further information and resources should be directed to and will be fulfilled by the lead contact, Kseniia Boxleitner (boxleitner@gea.mpg.de).

### Materials availability

This study neither produced any new materials and reagents nor reports original code. The paper includes existing published data that are analyzed together with the data produced by this study, listed in the key resources table.

### Data and code availability

Data produced by the archaeobotanical analysis necessary to interpret and replicate results are reported in the Main Text, Supplemental Tables, and Supplemental Data. This paper does not report original code. Any additional information required to reanalyze the data reported in this paper is available from the lead contact upon request.

## Acknowledgments

Field work at the archaeological sites was provided in the framework of RSF 24-78-10127, and the description of the site’s background was supported by 10.13039/501100001665the ANR project PaleoCALM (ANR-23-CE27-0019). The plant macrofossil analysis and radiocarbon dating were covered by the 10.13039/501100000781European Research Council, grant number 851102, awarded to Spengler. We thank Dr. Frank Kienast, local collaborators, and fieldwork teams for their support and fruitful discussions.

## Author contributions

Conceptualization, K.B., R.N.S., and S.S.; methodology, K.B., R.N.S., and S.S.; fieldwork, K.B., S.S., T.C., and V.A.; visualization, K.B.; laboratory work, K.B.; resources, R.N.S., S.S., T.C., A.A., and N.S.; writing – original draft, K.B.; writing – review and editing, K.B., R.N.S., S.S., V.A., T.C., A.A., and N.S.

## Declaration of interests

The authors declare no competing interests.

## STAR★Methods

### Key resources table

**Table:** 

REAGENT or RESOURCE	SOURCE	IDENTIFIER
**Deposited data**		

Archaeobotanical remains (plant macrofossils) from Surungur, Obishir V, and Kurteke sites; raw and analyzed data	This paper	Tables S2, S3, and S4
Chronometric data, analyzed data	This paper	Table S1

**Software and algorithms**		

IntCal20.14c model	Reimer et al., 2020^142^	https://c14.arch.ox.ac.uk/oxcal.html
Illustrator 2025	Adobe Inc.	https://www.adobe.com/products/illustrator.html

### Method details

#### Archaeobotanical analysis

Archaeobotanical samples for the current study were collected during four summer field seasons, spanning 2019-2022 (Figures 4, 5, and 6; Tables S2, S3, and S4). The number of samples varied between sites and excavation units (e.g. trenches and vertical walls), and the sampling strategy was picked accordingly (see Table 1 for detailed information). At Obishir V, targeted sampling was applied and focused on fire places and high-density ash features. At Surungur, the excavation area was split into equal squares to ensure the location of the recovered material and blanket sampling was applied, while additional targeted sampling focused on fire places. At Kurteke, a sample was taken from a fireplace in a test trench in 2019.

**Table 1:** Detailed sample information from the studied sites

Site name	Number of samples	Total volume, l	Sampling year	Sampling strategy
Obishir V	14	80.65	2018, 2019, 2021	Judgment sampling strategy was focused on fire places and ashy deposits
Surungur	91	897.95	2019, 2021, 2022	Judgment sampling strategy was focused on fire places and ashy features throughout layer 1, while a blanket sampling strategy was applied for the layers 2 - 3
Kurteke	1	4	2019	Test trench, targeted sampling of a fire place

In total, 106 samples were taken from the three sites, resulting in 982.6 liters of sediment. During fieldwork, the collected samples for archaeobotanical study were processed using a standard bucket flotation method with sieve sizes of 1.4 mm for heavy fraction and 0.35 mm for light fraction (after^143^^,^^144^). After flotation, each sample, consisting of light and heavy fractions, was individually packaged into a cotton bag and air-dried in the shade. After drying, heavy fraction material was dry screened in the field; all charred plant material, including charcoal, seeds, and fruit stone fragments were picked out with tweezers and individually packed for further laboratory analysis. The remaining material, including sediments, stone, and bone artifacts, was handed over to the appropriate specialists for further analysis. Light fractions of all samples and hand-sorted plant remains were sent to the paleoethnobotanical laboratory of the Max Planck Institute of Geoanthropology, Jena, Germany.

Once in the lab, samples were passed through nested U.S. geological sieves (2.0, 1.4, 1.0, and 0.5 mm) for ease of sorting. Material smaller than 0.5 mm was not sorted. Carbonized wood fragments larger than 2.00 mm were counted. Seeds and seed fragments were separated from all sieve units. Only charred seeds were systematically collected, on the assumption that uncarbonized seeds could be intrusive. All of the identified taxa are presented in Tables 2-4, and photos of key taxa were taken with a Keyence VHX6000 digital microscope and presented in Figures 4, 5, and 6. The category ‘Cerealia’ was used when a grain was too damaged and/or too small (less than one third of its original size) to distinguish between wheat and barley.

#### Radiocarbon dating

Plant remains for radiocarbon dating were sent to Woods Hole Oceanographic Institution (NOSAMS), USA, the Curt-Engelhorn-Center Archaeometry GmbH, Mannheim, Germany, and to the Center for Applied Isotope Studies, University of Georgia, USA (Table S1). The reported dates were calibrated using IntCal20.14c.^142^

### Quantification and statistical analysis

Original archaeobotanical raw data are provided in Supplemental Tables S2, S3, and S4, with quantified results reported and interpreted in the main text. The numbers of identified plant fragments (“n”) are specified both in the text and in Tables S2, S3, and S4. The Minimum Number of Individuals (MNI), used to estimate the number of whole domesticated grains and their fragments larger than half of a seed, is also reported as “n” in the text. The MNI method was not applied to nutshell fragments and other plant remains.

Thirteen charred plant macrofossils and one charcoal fragment were directly dated to refine the chronology of the site units. Analyses were carried out at four radiocarbon laboratories: the Curt-Engelhorn-Center Archaeometry GmbH (Germany), Woods Hole (USA), CAIS (USA), and Oxford (UK) radiocarbon laboratories. Calibrated ages are reported as median probabilities of the 95.4% (2σ) probability range in cal BCE (Figure 3; Table S1).

## Supplemental information

Document S1. Tables S1, S2, S4, S5, and supplemental references

Table S3. Archaeobotanical remains from Surungur rockshelter, related to Figures 3 and 5
