# Supplementary material for: Archaeobotanical investigations at high-elevation sites of the Pamir Mountains and fergana foothills
Source: iScience. 2025 Dec 5;29(1):114349. doi: 10.1016/j.isci.2025.114349 (PMC12775865; doi:10.1016/j.isci.2025.114349)
Supplement: Document S1. Tables S1, S2, S4, S5, and supplemental references [file mmc1.pdf]

iScience, Volume *29*

Supplemental information

Archaeobotanical investigations at high-elevation sites of the Pamir Mountains and fergana foothills

Kseniia Boxleitner, Robert N. Spengler III, Valentina Alekseitseva, Temirlan Chargynov, Aida Abdykanova, Nuritdin Sayfuloev, and Svetlana Shnaider

Supplementary Material

Table S1. Results of the direct radiocarbon dating of plant macrofossils from the Obishir V, Surungur, and Kurteke. OS- marks Woods Hole radiocarbon lab, octothorpe indicates CAIS lab, OxA stands for the Oxford Radiocarbon laboratory, asterisk marks the Curt-Engelhorn-Center Archaeometry GmbH, Mannheim, Germany. Related to Table 1, Figure 3.

	General data		Uncalibrated age		Calibrated age (cal. BCE)		
**Site name**	**Lab ID**	**Specimen**	**Age**	**Age error**	**1 Sigma probability (68.3 %)**	**2 Sigma probability (95.4%)**	**Median probability**
Obishir V	OxA-40179	*Triticum sp.*	1548	19	535-569 CE	528-579 CE	553 CE
	OxA-40180	*Hordeum sp.*	1615	18	419-436 CE	415-482 CE	448 CE
Surungur	OS-164836	*Cerealia*	3350	25	1644-1608	1691-1538	1614
	78279*	*Setaria italica*	3383	21	1728-1626	1741-1617	1679
	69948#	*Panicum miliaceum*	3680	30	2135-2080	2143-2007	2076
	OS-173985	*Panicum miliaceum*	3820	65	2349-2195	2465-2131	2298
	OS-173986	*Juglans regia*	6160	35	5127-5045	5211-5006	5108
	OS-173987	*Pistacia vera*	6630	45	5571-5528	5626-5481	5553
	69340#	*Fabaceae*	6880	30	5788-5724	5841-5711	5760
	69948#	*Panicum miliaceum*	3680	30	2135-2080	2143-2007	2076
	69947#	*Juglans regia*	6930	30	5837-5753	5851-5731	5802
	69946#	*Hordeum type*	6840	35	5743-5703	5795-5641	5720
Kurteke	OS-164817	*Potentilla sp.*	8850	40	8014-7940	8211-7785	7998
	OS-164817	*charcoal*	8940	45	8246-8170	8150-7957	8053

Table S2. Identified plant remains from Obishir V and Kurteke rockshelters. Red color text indicates directly radiocarbon dated specimens. Related to Figures 3, 4, and 6.

	Sampling Year	2018	2019	2021											2019	Total
	Site	Obishir V													Kurteke	
	layer	1	2	2	2	2	2,3	2,3	2,4	2,4	2,4	3	3	3	1	
	volume (l)	4,95	10	6,8	10,1	7,2	4,5	-	8,6	4,2	7,2	4,5	6,5	6,1	4	
Identification	Sample name	1 Sq T 8-9	N3835	4877	4833	4896	4922	5324	5030	5031	4924	4726	5138	5124		
Grain Parts	*Triticum aestivum* rachis	4														**4**
	Triticum durum rachis	7														**7**
	Barley Rachis	5														**5**
	Wheat glume base	4														**4**
	Cerealia	28														**28**
Domestic Grains	*Hordeum vulgare* var. *vulgare*	3														**3**
	Hordeum vulgare var. *nudum*	**4**														**4**
	Triticum aestivum/durum	**12**														**12**
	Setaria italica	2														**2**
Fruits and Nuts	*Juglans regia*	8	3													**11**
	Rosa sp.	2														**2**
Amaranthaceae	Amaranthaceae	6					33		3		2		12	3		**59**
	Chenopodium iljinii														1	**1**
	Chenopodium sp.	869														**869**
Asteraceae	Asteraceae	4														**4**
	Onopordum acanthium	3														**3**
	Xanthium strumarium	1														**1**
Boraginaceae	*Lithospermum arvense*	2												1		**3**
Brassicaceae	Brassicaceae	3														**3**
	Euclidium syriacum				1											**1**
Caryophyllaceae	Caryophyllaceae	3		1												**4**
	Vaccaria hispanica										1					**1**
Convolvolaceae	Convolvolaceae	1														**1**
Cupressaceae	*Juniperus* sp.	1														**1**
Cyperaceae	*Carex stenophylla* subsp. *stenophylloides*														5	**5**
Fabaceae	Fabaceae type small	24														**24**
	Fabaceae	1		2											6	**9**
	Trigonella			1		1			2							**4**
Lamiaceae	*Ajuga* sp.	1														**1**
Malvaceae	*Malva* sp.	6														**6**
Poaceae	Poaceae	2														**2**
	Poaceae type small	3													13	**16**
	Pooid	2														**2**
	Setaria (wild)	5														**5**
	Stipa sp.	13														**13**
Polygonaceae	Polygonaceae	2														**2**
	Polygonum sp.	2														**2**
Rosaceae	*Potentilla/ Fragaria*	8					1								**292**	**301**
Rubiaceae	*Galium* sp.	5	1													**6**
Solanaceae	*Hyoscyamus niger*	1														**1**
Thymelaeaceae	*Thymelaea passerina*	1													1	**2**
Unidentifiable	Unidentifiable Seed Fragments	430													64	**494**
Charcoal		NC	NC	1020	2265	2487	436	-	4538	3182	1005	38	69	136	**1292**	**16468**
Egg shells				6	21	11	9	2	34	48	16	-	4	6		
**Totals**	**without Unidentifiable Fragments**	**1048**	**4**	**4**	**1**	**1**	**34**	**0**	**5**	**0**	**3**	**0**	**12**	**4**	**318**	**1434**

Table S4. Charcoal, bone, and snail fragments from additional ten samples, that yield no plant macrofossil remains from the Surungur rockshelter. Related to Figures 7 and 8.

Excavation year	2021		2022								
Layer	1.2	1.2	3.1		3.2	3.2	3.2	3.4	3.4	3.5	
square	J16-3/4	L16-3/4		Q 50	P 50	Q 51	Q 50	P 51	P 51	P 51	**Totals**
charcoal (>2 mm)	28	7	1	23	19	32		3	28	8	**149**
bone			1		1	1					**3**
egg shell	2	1									**3**

Table S5. Overview of millet finds along the IAMC and adjacent regions discussed in the paper. Asterisk marks radiocarbon dates originating from associated archaeological layers/plant macrofossils, caret indicates dates obtained from human individuals, while tilde indicates dating of charcoal, human and sheep collagen. Related to Figures 1, 2.

Site	Present day country	Calibrated date (BCE)	Plant identification	Type of data	Reference
Karuo	China	2900-2600	broomcorn millet	archaeobotany	Lu[1]
Dali	Kazakhstan	2850-2500*	foxtail millet	pottery impressions	Endo et al.[2]
Ayituohan I	China	2836-2490^	millets	isotopes	Qu et al.[3]
Dali	Kazakhstan	2705-2545~	millets	isotopes	Hermes et al.[4]
Karuo	China	2700-2300	foxtail millet	archaeobotany	d'Alpoim Guedes et al.[5]
Baiyangcun	China	2868-2573	broomcorn and foxtail millets	archaeobotany	Dal Martello et al.[6]
Pethpuran Teng	India	2500-1950	broomcorn millet	archaeobotany	Yatoo et al.[7]
Surungur	Kyrgyzstan	2465-2131	broomcorn millet	archaeobotany	current study
Begash	Kazakhstan	2460-2150	broomcorn millet	archaeobotany	Frachetti et al.[8]
Argyzhal-3	Kyrgyzstan	2460-2204^	likely broomcorn millet	isotopes	Motuzaite Matuzeviciute et al.[9]
Adji Kui	Turkmenistan	2200*	broomcorn millet	archaeobotany	Spengler et al.[10]
Tongtian Cave	China	2199-1981	broomcorn millet	archaeobotany	Zhou et al.[11]
Togolok	Turkmenistan	2197-1983	broomcorn millet	archaeobotany	Billings et al.[12]
Surungur	Kyrgyzstan	2143-2007	broomcorn millet	archaeobotany	current study
Ghal e-Ben	Iran	2141-1951	broomcorn millet	archaeobotany	Huang et al.[13]
Xiaohe	China	2011-1756	broomcorn millet	archaeobotany	Flad et al.[14]
Dali	Kazakhstan	1850-1550*	broomcorn and foxtail millets	pottery impressions	Endo et al.[2]
Kyzyl-Bulak I	Kazakhstan	1754-1541^	millets	isotopes	Motuzaite Matuzeviciute et al.[15]
Surungur	Kyrgyzstan	1741-1617	foxtail millet	archaeobotany	current study
Adunqiaolu	China	1616-1282*	broomcorn and foxtail millets	archaeobotany	Tian et al.[16]
Oi-Dzhailau-VII	Kazakhstan	1608-1428^	millets	isotopes	Motuzaite Matuzeviciute et al.[15]
Tasbas	Kazakhstan	1400*	foxtail millet	archaeobotany	Spengler[17]
Uch-Kurbu	Kyrgyzstan	1366-1124*	foxtail and broomcorn millets	archaeobotany	Motuzaite Matuzeviciute et al.[9]
Chap I	Kyrgyzstan	1065-825*	foxtail and broomcorn millets	archaeobotany	Motuzaite Matuzeviciute et al.[18]

Supplemental references

1. Lu, H. (2023). Local millet farming and permanent occupation on the Tibetan Plateau. Sci China Earth Sci *66*, 430–434. https://doi.org/10.1007/s11430-022-1018-7.
2. Endo, E., Shoda, S., Frachetti, M., Kaliyeva, Z., Kiyasbek, G., Zhuniskhanov, A., Liu, X., and Dupuy, P.D. (2023). Pottery Impressions Reveal Earlier Westward Dispersal of Foxtail Millet in Inner Asian Mountain Corridor. Agronomy *13*, 1706. https://doi.org/10.3390/agronomy13071706.
3. Qu, Y., Hu, X., Wang, T., and Yang, Y. (2020). Early interaction of agropastoralism in Eurasia: new evidence from millet-based food consumption of Afanasyevo humans in the southern Altai Mountains, Xinjiang, China. Archaeol Anthropol Sci *12*, 195. https://doi.org/10.1007/s12520-020-01094-2.
4. Hermes, T.R., Frachetti, M.D., Doumani Dupuy, P.N., Mar'yashev, A., Nebel, A., and Makarewicz, C.A. (2019). Early integration of pastoralism and millet cultivation in Bronze Age Eurasia. Proceedings of the Royal Society B: Biological Sciences *286*, 20191273. https://doi.org/10.1098/rspb.2019.1273.
5. d'Alpoim Guedes, J., Lu, H., Li, Y., Spengler, R.N., Wu, X., and Aldenderfer, M.S. (2014). Moving agriculture onto the Tibetan plateau: the archaeobotanical evidence. Archaeol Anthropol Sci *6*, 255–269. https://doi.org/10.1007/s12520-013-0153-4.
6. Dal Martello, R., Min, R., Stevens, C., Higham, C., Higham, T., Qin, L., and Fuller, D.Q. (2018). Early agriculture at the crossroads of China and Southeast Asia: Archaeobotanical evidence and radiocarbon dates from Baiyangcun, Yunnan. J Archaeol Sci Rep *20*, 711–721. https://doi.org/10.1016/j.jasrep.2018.06.005.
7. Yatoo, M.A., Spate, M., Betts, A., Pokharia, A.K., and Shah, M.A. (2020). New evidence from the Kashmir Valley indicates the adoption of East and West Asian crops in the western Himalayas by 4400 years ago. Quaternary Science Advances *2*. https://doi.org/10.1016/j.qsa.2020.100011.
8. Frachetti, M.D., Spengler, R.N., Fritz, G.J., and Mar'yashev, A.N. (2010). Earliest direct evidence for broomcorn millet and wheat in the central Eurasian steppe region. Antiquity *84*, 993–1010. https://doi.org/10.1017/S0003598X0006703X.
9. Motuzaite Matuzeviciute, G., Ananyevskaya, E., Sakalauskaite, J., Soltobaev, O., and Tabaldiev, K. (2022). The integration of millet into the diet of Central Asian populations in the third millennium BC. Antiquity *96*, 560–574. https://doi.org/10.15184/aqy.2022.23.
10. Spengler, R.N., de Nigris, I., Cerasetti, B., Carra, M., and Rouse, L.M. (2018). The breadth of dietary economy in Bronze Age Central Asia: Case study from Adji Kui 1 in the Murghab region of Turkmenistan. J Archaeol Sci Rep *22*, 372–381. https://doi.org/10.1016/j.jasrep.2016.03.029.
11. Zhou, X., Yu, J., Spengler, R.N., Shen, H., Zhao, K., Ge, J., Bao, Y., Liu, J., Yang, Q., Chen, G., et al. (2020). 5,200-year-old cereal grains from the eastern Altai Mountains redate the trans-Eurasian crop exchange. Nat Plants *6*, 78–87. https://doi.org/10.1038/s41477-019-0581-y.
12. Billings, T.N., Cerasetti, B., Forni, L., Arciero, R., Dal Martello, R., Carra, M., Rouse, L.M., Boivin, N., and Spengler, R.N. (2022). Agriculture in the Karakum: An archaeobotanical analysis from Togolok 1, southern Turkmenistan (ca. 2300–1700 B.C.). Front Ecol Evol *10*. https://doi.org/10.3389/fevo.2022.995490.
13. Huang, Y., Deng, Z., Nashli, H.F., Fuller, D.Q., Wu, X., and Safari, M. (2023). The early adoption of East Asian crops in West Asia: rice and broomcorn millet in northern Iran. Antiquity *97*, 674–689. https://doi.org/10.15184/aqy.2023.42.
14. Flad, R., Shuicheng, L., Xiaohong, W., and Zhijun, Z. (2010). Early wheat in China: Results from new studies at Donghuishan in the Hexi Corridor. Holocene *20*, 955–965. https://doi.org/10.1177/0959683609358914.
15. Motuzaite Matuzeviciute, G., Lightfoot, E., O'Connell, T.C., Voyakin, D., Liu, X., Loman, V., Svyatko, S., Usmanova, E., and Jones, M.K. (2015). The extent of cereal cultivation among the Bronze Age to Turkic period societies of Kazakhstan determined using stable isotope analysis of bone collagen. J Archaeol Sci *59*, 23–34. https://doi.org/10.1016/j.jas.2015.03.029.
16. Tian, D., Festa, M., Cong, D., Zhao, Z., Jia, P.W., and Betts, A. (2021). New evidence for supplementary crop production, foddering and fuel use by Bronze Age transhumant pastoralists in the Tianshan Mountains. Sci Rep *11*, 13718. https://doi.org/10.1038/s41598-021-93090-2.

17. Spengler, R.N. (2015). Agriculture in the Central Asian Bronze Age. J World Prehist *28*, 215–253. https://doi.org/10.1007/s10963-015-9087-3.
18. Motuzaite Matuzeviciute, G., Tabaldiev, K., Hermes, T., Ananyevskaya, E., Grikpedis, M., Luneau, E., Merkyte, I., and Rouse, L.M. (2020). High-Altitude Agro-Pastoralism in the Kyrgyz Tien Shan: New Excavations of the Chap Farmstead (1065–825 cal b.c.). J Field Archaeol *45*, 29–45. https://doi.org/10.1080/00934690.2019.1672128.
